# Tungsten and Cobalt in Fallon, Nevada: Association with Childhood Leukemia

**DOI:** 10.1289/ehp.10614

**Published:** 2008-05

**Authors:** John Schell, Michael Pardus

**Affiliations:** ENTRIX, Inc., Houston, Texas, E-mail: jschell@entrix.com; Arcadis-BBL, Pittsburgh, Pennsylvania, E-mail: Mike.Pardus@Arcadis-US.com

In their article, Sheppard et al. (2007) suggested that the “results in Fallon [Nevada] suggest a temporal correspondence between the onset of excessive childhood leukemia and elevated levels of tungsten and cobalt.” Although the authors reported some interesting findings from their dendrochemistry (tree ring) analysis, the results, as presented, do not support their conclusion. In fact, if the data they report demonstrate anything, it is that the levels of tungsten in the environment are not causally associated with the cases of leukemia.

Sheppard et al.’s (2007) primary premise is that levels of tungsten increased in Fallon relative to selected comparison towns beginning in the mid-1990s, which the authors contend predates the “1997 onset” of the increased incidence of acute lymphocytic leukemia (ALL) in Churchill County. In fact, when the appropriate comparison is made, the data of Sheppard et al. show that the purported increase in environmental tungsten in Fallon occurred long after the onset of these leukemia cases (i.e., after 2001). Thus, the data they gathered supports the conclusion of the Agency for Toxic Substances and Disease Registry (2003) that the evidence does not support any link between tungsten and these leukemia cases.

Unfortunately, Sheppard et al. (2007) did not provide the actual data in their article, and they did not include error bars or standard deviations for the data points. Regardless of these defects in data presentation, careful interpretation of the graphic representations of the data yields several important insights [the tungsten concentrations listed here are approximate and were obtained from interpolation of the data points from the figures of Sheppard et al. (2007)].

First, tree ring tungsten concentrations in Fallon cottonwoods ranged from 40 to 70 ppm between 1989 and 2000, and then increased to 180 ppm in the 2001–2004 time period. Sweet Home, Oregon, cottonwoods followed a similar pattern, ranging from 50 to 75 ppm between 1989 and 2000 and increasing to 110 ppm for the 2001–2004 period. Thus, no significant increase in tungsten in cottonwood tree rings was observed at either location until the 2001–2004 period, well after the “onset” of the leukemia cases; 12 of the 15 leukemia cases (80%) had been diagnosed by the end of 2000 [Centers for Disease Control and Prevention (CDC) 2003].

Second, it appears that the “comparison town” data comprise the Douglas-fir data from Crawfordsville, Oregon, and data on Douglas-firs and cottonwoods from Sweet Home. Sheppard et al. (2007) acknowledged that “temporal variability of tungsten is higher in the cottonwoods than in the Douglas-firs,” and Douglas-firs exhibit “damped temporal variability.” This is likely to be at least partially due to physiologic differences of tree species (Sheppard et al. 2007). Comparing tungsten levels in responsive cottonwoods in Fallon to groups of trees from comparison towns that included less responsive Douglas-firs is an “apples-to-oranges” comparison, and any differences are more likely related to the differences in tree species than to different levels of environmental tungsten. This uncertainty is further exacerbated by comparing trees from vastly different environments—the temperate, agricultural areas of northwestern Oregon and the high desert of western Nevada.

Third, Sweet Home cottonwoods exhibited an average of 62 ppm tungsten between 1989 and 2000. Fallon cottonwoods had an average of 60 ppm during this same period. The purported temporal variability between the two locations is nonexistent when like species are compared.

Finally, according to Sheppard et al. (2007), the 1989–1996 period represents two time periods that “predate the 1997 onset of excessive leukemia ….” Yet, compared with those in Fallon, the Sweet Home cottonwoods exhibited slightly higher tungsten concentrations over the 1989–1996 period. Thus, according to the data of Sheppard et al. (2007), environmental tungsten was actually lower in Fallon than in Sweet Home during the period leading up to the diagnosis of the Fallon leukemia cases.

In summary, a critical evaluation of the data leads to a radically different conclusion than that presented by Sheppard et al. (2007). Assuming that the data presented in the article are correct and reflective of environmental conditions in the Fallon area, the data indicate that the purported increase in tungsten levels (if in fact any increase occurred) occurred well after the “onset of the excessive childhood leukemia” and was not unique to this town. This interpretation is consistent with, and further supports, the conclusions that tungsten was not associated with the ALL cluster reached by the CDC in their *Cross-Sectional Exposure Assessment in Fallon* (CDC 2003; Rubin et al. 2007).

## References

[b1-ehp0116-a00196] Agency for Toxic Substances and Disease Registry (2003). Health Consultation. Churchill County Tap Water. Fallon Leukemia Project. Fallon, Churchill County, Nevada. http://www.atsdr.cdc.gov/sites/fallon/fallon-leukemia062403-ne-tapwater.pdf.

[b2-ehp0116-a00196] CDC (2003). A Cross-Sectional Exposure Assessment of Environmental Exposures in Churchill County, Nevada. Final Report. http://www.cdc.gov/nceh/clusters/Fallon/study.htm.

[b3-ehp0116-a00196] Rubin CS, Holmes AK, Belson MG, Jones RL, Flanders WD (2007). Investigating childhood leukemia in Churchill County, Nevada. Environ Health Perspect.

[b4-ehp0116-a00196] Sheppard PR, Speakman RJ, Ridenour G, Witten ML (2007). Temporal variability of tungsten and cobalt in Fallon, Nevada. Environ Health Perspect.

